# FSP1^+^ fibroblast subpopulation is essential for the maintenance and regeneration of medullary thymic epithelial cells

**DOI:** 10.1038/srep14871

**Published:** 2015-10-08

**Authors:** Lina Sun, Chenming Sun, Zhanfeng Liang, Hongran Li, Lin Chen, Haiying Luo, Hongmei Zhang, Pengbo Ding, Xiaoning Sun, Zhihai Qin, Yong Zhao

**Affiliations:** 1 State Key Laboratory of Biomembrane and Membrane Biotechnology, Institute of Zoology, Chinese Academy of Sciences, Beijing, China; 2 Key Laboratory of Protein and Peptide Pharmaceuticals, Institute of Biophysics, Chinese Academy of Sciences, Beijing, China

## Abstract

Thymic epithelial cells (TECs) form a 3-dimentional network supporting thymocyte development and maturation. Besides epithelium and thymocytes, heterogeneous fibroblasts are essential components in maintaining thymic microenvironments. However, thymic fibroblast characteristics, development and function remain to be determined. We herein found that thymic non-hematopoietic CD45^-^FSP1^+^ cells represent a unique Fibroblast specific protein 1 (FSP1)^—^fibroblast-derived cell subset. Deletion of these cells in FSP1-TK transgenic mice caused thymus atrophy due to the loss of TECs, especially mature medullary TECs (MHCII^high^, CD80^+^ and Aire^+^). In a cyclophosphamide-induced thymus injury and regeneration model, lack of non-hematopoietic CD45^-^FSP1^+^ fibroblast subpopulation significantly delayed thymus regeneration. In fact, thymic FSP1^+^ fibroblasts released more IL-6, FGF7 and FSP1 in the culture medium than their FSP1^-^ counterparts. Further experiments showed that the FSP1 protein could directly enhance the proliferation and maturation of TECs in the *in vitro* culture systems. FSP1 knockout mice had significantly smaller thymus size and less TECs than their control. Collectively, our studies reveal that thymic CD45^-^FSP1^+^ cells are a subpopulation of fibroblasts, which is crucial for the maintenance and regeneration of TECs especially medullary TECs through providing IL-6, FGF7 and FSP1.

The thymus is a primary lymphoid organ, which is essential for T cell development and maturation. The unique thymic microenvironment consists of complex cellular composition including hematopoietic and non-hematopoietic cells^1^,^2^. Among all thymic cell components, thymic epithelial cells (TECs) are of the most significance to provide highly specialized microenvironments and essential instructive signals for the functional and self-tolerant T cell maturation from progenitor cells in the thymus^3^,^4^. TECs are roughly divided into two major subsets: cortical TECs (cTECs) and medullary TECs (mTECs), simply based on the localization in the thymus and distinctive cell surface markers^5^,^6^. The complete partitioning into mature cTECs and mTECs requires reciprocal instructive signals from developing thymocytes, a bidirectional interaction known as “thymic crosstalk”^7^,^8^,^9^. Fibroblasts, a group of heterogeneous multifunctional cells of mesenchymal origin, produce many immune modulators and play an important regulatory role in inflammation, wound healing, and tissue fibrosis^10^,^11^,^12^,^13^. It is reported that fibroblastic cell lines supported the development of the mouse thymus anlage in organ culture system^14^. Fibroblasts are a significant regulator in promoting early thymocyte development and TEC development, proliferation and regeneration^15^,^16^,^17^,^18^. Mesenchyme was found to be essential for TEC proliferation during embryogenesis through the production of fibroblast growth factor 7 (FGF7, also named as keratinocyte growth factor; KGF) and FGF10^17^,^19^,^20^. Thus, the development and maturation of TECs critically depend on the complicate microenvironments, mainly offered by residual surrounding cells such as immune cells and fibroblasts.

Fibroblast heterogeneity has been appreciated for several decades^21^,^22^,^23^, but its biological significance and the basis for cellular diversity remain uncertain. At present, ER-TR7 and MTS-15 are considered as specific markers for thymic fibroblasts^16^,^24^. However, markers for thymic fibroblasts are easily confusing with mesenchymal cells^25^. Fibroblast-specific protein 1 (FSP1, also named as S100A4), one member of the S100 superfamily of cytoplasmic calcium-binding proteins, is predominately expressed in fibroblasts but not in epithelial cells in organs undergoing tissue remodeling like skin, kidney, lung, and heart, as well as in some other cell types in certain conditions^26^,^27^,^28^,^29^. The presence, characteristics and biological significance of non-hematopoietic FSP1^+^ cells in the thymus have not been determined. In the present study, using FSP1-GFP reporter mice, FSP1^+^ cells-deleting mice (FSP1-thymidine kinase (TK) transgenic mice), FSP1 knockout (FSP1KO) mice, and many experimental mouse models, we tried to investigate the characteristics and biological significance of non-hematopoietic FSP1^+^ cells in the thymus. We found that a subpopulation of fibroblasts but no epithelial cells express FSP1 in the thymus. A series of *in vivo* and *in vitro* studies indicated that non-hematopoietic CD45^−^FSP1^+^ fibroblast subpopulation plays an important nursing role on TEC maintenance and regeneration via providing IL-6, FGF7 and FSP1. The present study shed lights on the critical roles of FSP1^+^ fibroblast subset and FSP1 on mTEC development.

## Results

### Thymic CD45^-^FSP1^+^ cells are a subpopulation of fibroblasts

**Figure 1:**
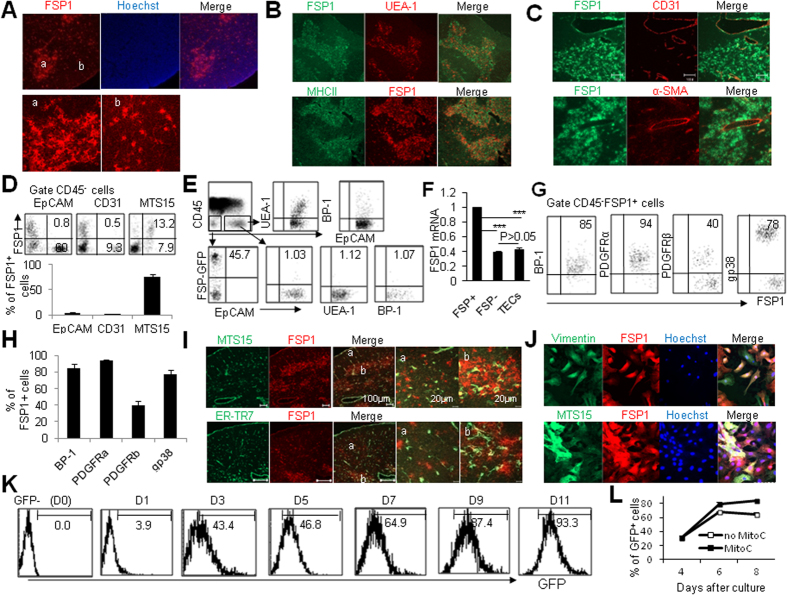
Characteristics of FSP1 expression in the thymus. Frozen thymic sections from 6-8-wks-old WT mice were co-stained with FSP1 and Hoechst 33342 (**A**) or UEA-1 and MHCII (**B**) or CD31 and α-SMA (**C**). (**D**) Phenotypic characterization of FSP1 *vs* EpCAM, CD31 and MTS15 expression in the gated thymic CD45^−^ cells of FSP1-GFP reporter mice was shown. (**E**) Representative flow cytometry staining of UEA-1, BP-1, and FSP1-GFP^+^ cells among the gated CD45^−^EpCAM^−^ and CD45^−^EpCAM^+^ (UEA1^+^ and BP-1^+^) cells in FSP1-GFP mice. (**F**) The mRNA expression of FSP1 in TECs, FSP1^+^ and FSP1^−^ fibroblasts as determined by real-time PCR. (**G**) Representative flow cytometry staining and the percentage of BP-1, PDGFRα, PDGFRβ and gp38 cells among the gated CD45^−^FSP1^+^ cells in FSP1-GFP mice. (**H**) The percentage of BP-1, PDGFRα, PDGFRβ and gp38 cells among CD45^−^FSP1^+^ cells in the thymus of FSP1-GFP mice. (**I**) Staining of frozen thymic sections from 6–8 wk WT mice with FSP1 and MTS15 (the upper panel) and ER-TR7 (the lower panel) was shown. (**J**) Cultured primary thymic fibroblasts were stained with FSP1 and vimentin or MTS15. (**K**) FSP1-GFP expression in thymic fibroblasts was assayed at different culture time points. (**L**) Percentage of FSP1^+^ cells in primary thymic fibroblasts with or without 10 μg/ml mitomycin C treatment for 4 hours. Representative results are shown from one of three independent experiments performed.

FSP1 was originally recognized as a specific marker for fibroblasts^26^. However, it was recently challenged by the observation showing the expression of FSP1 in other cells in inflammatory situations^30^. Considering the fibroblast heterogeneity and the differences of fibroblasts in different organs^16^,^21^,^22^,^23^, we firstly investigated the expression pattern of FSP1 in different cell types in the thymus using immunohistochemical staining assays. Immunofluorescence analysis of adult mouse thymus sections with anti-FSP1 antibody revealed specific and extensive staining (Fig. 1A). The staining patterns of FSP1 in thymic medulla and cortex regions were different. FSP1 was expressed intensively and distributed clusteredly in medulla area, whereas FSP1 in cortex area was less and point shape distribution (Fig. 1A). Co-staining of FSP1 and mTEC marker UEA-1 or MHCII showed majority of FSP1^+^ cells were located in thymic medullary area (Fig. 1B). Because CD31, known as platelet/endothelial cell adhesion molecule–1, is widely recognized and frequently used as a sensitive and relatively specific immunohistochemical marker of endothelial cells and thereby vascular neoplasia^31^, we thus investigated whether CD31^+^ cells express FSP1 in the thymus. As shown in Fig. 1C, no CD31^+^ cells were co-stained with FSP1. In addition, no FSP1^+^ cells in the thymus express α-smooth muscle actin (α-SMA) (Fig. 1C), one of the mesenchymal markers^32^. To further determine the expression pattern of FSP1 in the thymus, we used FSP1-GFP reporter mice^33^, in which the FSP1 promoter drives green fluorescent protein (GFP) expression, to mark and trace FSP1^+^ cells. No CD45^−^FSP1^+^ cells in the thymus express CD31 in WT and FSP-GFP reporter mice (Fig. 1C,D). Clearly, TECs including CD45^−^EpCAM^+^ TECs, CD45^−^EpCAM^+^UAE-1^+^ mTECs, and CD45^−^EpCAM^+^BP-1^+^ cTECs of FSP-GFP reporter mice did not express detectable FSP1 as determined by flow cytometry (Fig. 1D,E). The undetectable expression of FSP1 in TECs was also confirmed by real-time PCR with FSP1^+^ and FSP1^−^ fibroblasts as positive and negative control, respectively (Fig. 1F). The poor FSP1 expression in TECs was in line well with a recent report^34^. However, part of CD45^−^FSP1^+^ cells isolated from FSP1-GFP reporter mice showed MTS15^+^, one marker for thymic fibroblasts^16^, as determined by flow cytometry (Fig. 1D). In addition, CD45^−^FSP1^+^ cells expressed thymic mesenchymal cell markers BP-1 and gp38, and the majority of CD45^−^FSP1^+^ cells expressed PDGFRα and PDGFRβ (Fig. 1G,H), indicating their mesenchymal origin. Furthermore, immunohistological staining of the thymus showed that only a small fraction of FSP1^+^ cells were co-localized with MTS15 and ER-TR7, respectively (Fig. 1I). More impressively, the distribution of MTS15^+^ and ER-TR7^+^ fibroblasts in the thymus was different from the distribution of FSP1^+^ cells (Fig. 1I). To further confirm whether thymic CD45^−^FSP1^+^ cells were fibroblasts, we cultured mouse primary thymic fibroblasts *in vitro*. They displayed classical spindle shape and expressed FSP1 (Suppl. Fig. 1A) and fibroblast markers vimentin, MTS15 and PDGFRα (Fig. 1J, Suppl. Fig. 1B). Furthermore, the cultured thymic FSP1^+^ cells did not express pan CK, CD11b, UEA-1, CD11c, and F4/80, markers for other cell types (Suppl. Fig. 1C). All these results provided evidence that thymic CD45^−^FSP1^+^ cells were not TECs, endothelial cells or perivascular smooth muscle cells, but likely were a unique subgroup of fibroblasts, which was different from the known MTS15^+^ and ER-TR7^+^ fibroblasts^16^.

In order to investigate whether FSP1^−^ thymic fibroblasts could transfer into FSP1^+^ cells or vise verse, we cultured the sorted FSP1-GFP^−^ and FSP1-GFP^+^ thymic fibroblasts of FSP1-GFP reporter mice *in vitro*. We surprisingly found that the sorted FSP1-GFP^—^fibroblasts could generally change into FSP1-GFP^+^ cells in a time-dependent manner (Fig. 1K). Although FSP1-GFP^−^ fibroblasts could turn into FSP1-GFP^+^ cells, while FSP1-GFP^+^ cells stayed FSP1 positive status (Supplemental Fig. 1D,E). Moreover, this change of FSP1 expression is independent on cell proliferation. Cell proliferation was inhibited when the isolated thymic fibroblasts were treated with mitomycin C (MitoC) for 4 hrs and were then cultured for following 4 days (Suppl. Fig. 1F). When thymic fibroblasts of FSP1-GFP mice were treated with MitoC for 4 hrs after 4 days culture, these MitoC-treated FSP1-GFP^−^ thymic fibroblasts could also turn into FSP1-GFP^+^ cells efficiently as cells without MitoC treatment (Fig. 1L). In addition, thymic FSP1-GFP^−^ and FSP1-GFP^+^ fibroblasts were different in their cell size. Thymic FSP1-GFP^−^ fibroblasts were smaller than FSP1-GFP^+^ fibroblasts as determined by their forward scatter signal when cells were assayed by flow cytometry (data not shown). Thus, thymic FSP1^+^ fibroblasts were a fraction of thymic fibroblasts which could be derived from FSP1^−^ fibroblasts.

### Deletion of thymic FSP1^+^ fibroblasts impairs thymic maintenance

**Figure 2:**
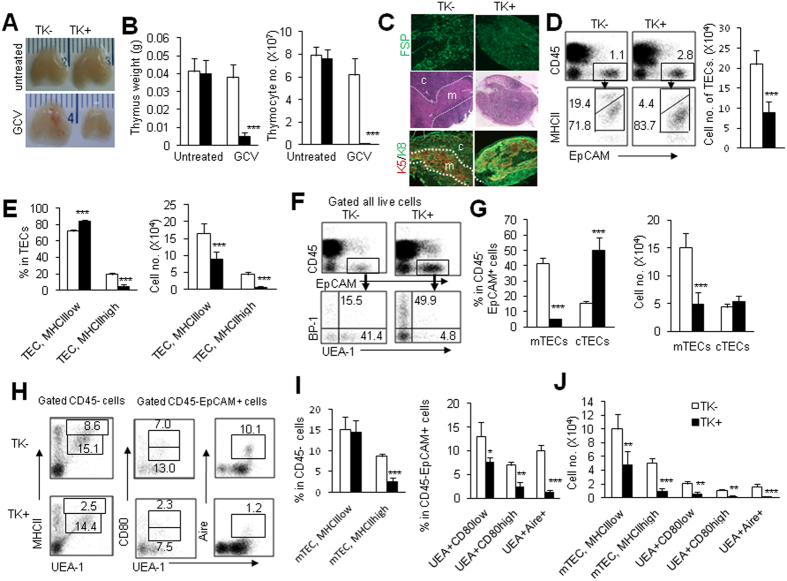
Deletion of FSP1^+^ cells dramatically altered thymus structure and cell composition. (**A**) Representative photographs of thymus organs in TK^−^ and TK^+^ transgenic mice with and without GCV treatment for 18 days were shown. (**B**) Thymus weight and total cell numbers of thymocytes in TK^−^ and TK^+^ mice with GCV treatment were summarized. (**C**) Thymic sections from GCV treated TK^−^ and TK^+^ mice were stained with FSP1 to determine the ablating efficiency (upper). The H&E and CK5/CK8 staining of thymic sections showed decreased area of thymic medullary region in TK^+^ mice than in TK^−^ mice after GCV treatment (middle and lower). (**D**) Representative FACS analysis of CD45^−^EpCAM^+^ TECs, MHCII^high^ and MHCII^low^ TECs in the isolated thymic cells was shown. The cell number of TECs in TK^+^ mice was less than in TK^−^ mice after GCV treatment. (**E**) The percentage and the total cell number of MHCII^high^ and MHCII^low^ TECs in GCV-treated TK^−^ and TK^+^ mice were summarized. Representative FACS profiles (**F**), and the percentage and total cell number (**G**) of mTECs and cTECs in CD45^−^EpCAM^+^ cells in GCV-treated TK^−^ and TK^+^ mice were shown. Phenotypic characterization (**H**) and the percentages (**I**) of MHCII^+^, CD80^+^ and Aire^+^ mTECs in the gated thymic CD45^−^ or CD45^−^EpCAM^+^ cells of TK^−^ and TK^+^ mice with GCV treatment. (**J**) The total cell numbers of CD45^−^EpCAM^+^UEA-1^+^MHCII^high^, CD45^−^EpCAM^+^UEA-1^+^CD80^high^, CD45^−^EpCAM^+^UEA-1^+^CD80^low^ and CD45^−^EpCAM^+^UEA-1^+^Aire^+^ cells in GCV-treated TK^−^ and TK^+^ mice. Representative results are shown from one of three independent experiments performed. Data were shown as mean ± SD (N = 5). *P < 0.05, **P < 0.01, ***P < 0.001 compared with TK^−^ mice.

To investigate the role of FSP1^+^ cells on the maintenance of TECs, FSP1-TK transgenic mice were used^35^,^36^, in which the expression of herpes simplex virus–derived thymidine kinase (TK) was under the control of the FSP1 promoter. Exogenous addition of oligonucleotide analogue, such as ganciclovir (GCV), phosphorylated product by TK could selectively delete proliferating FSP1^+^ cells *in vivo*^35^. In our current study, FSP1-TK mice (TK^+^) and wild-type (WT) control littermates (TK^−^) were treated with GCV for 18 days, thymic FSP1 expression was remarkably decreased in TK^+^ mice as determined by immunofluorescence (Fig. 2C). Compare to the GCV-treated TK^−^ control mice, GCV-treated TK^+^ mice had small thymus size, decreased thymus weight and total cell number of thymocyte (P < 0.001, Fig. 2A,B, and suppl. Fig. 2A). Thymocytes were severely affected with almost complete loss of thymocyte subsets (suppl. Fig. 2B). Thymic sections from TK^−^ and TK^+^ mice stained with H&E or mAbs against CK5 and CK8 revealed dramatic disruption of thymic structure with confusing thymic medulla and cortex region (Fig. 2C). The total cell number of thymic epithelial cells (CD45^−^EpCAM^+^) were also significantly decreased (Fig. 2D). Moreover, the percentage of TECs expressing high level of MHCII were decreased while MHCII^low^ TECs were relatively increased, although their cell number were significantly decreased (P < 0.001, Fig. 2E). We further analyzed TEC subsets by using UEA-1 and BP-1 as surface markers of mTEC and cTEC subpopulation respectively^6^. The results showed that both the ratio and cell number of mTECs (CD45^−^EpCAM^+^UEA-1^+^) were remarkably decreased in TK^+^ mice (P < 0.001, Fig. 2F,G). However, the total cell number of cTECs (CD45^−^EpCAM^+^BP-1^+^) was indistinguishable in TK^+^ mice and TK^−^ mice (Fig. 2G). The increased percentage of cTECs in TK^+^ mice was possibly caused by the relative decrease of mTEC components (Fig. 2F,G). Thus, deletion of thymic FSP1^+^ cells selectively affected mTECs in the thymus. Functional maturation of mTECs is marked by expressing high level of MHC II, CD80 and the transcriptional regulator Aire^6^,^37^. The mature mTECs (MHCII^high^, CD80^high^ and Aire^+^) were significantly decreased in GCV-treated TK^+^ mice compared with GCV-treated TK^−^ mice (Fig. 2H–J). These results suggested that deletion of FSP1^+^ cells selectively impaired the mature mTEC homeostasis.

**Figure 3:**
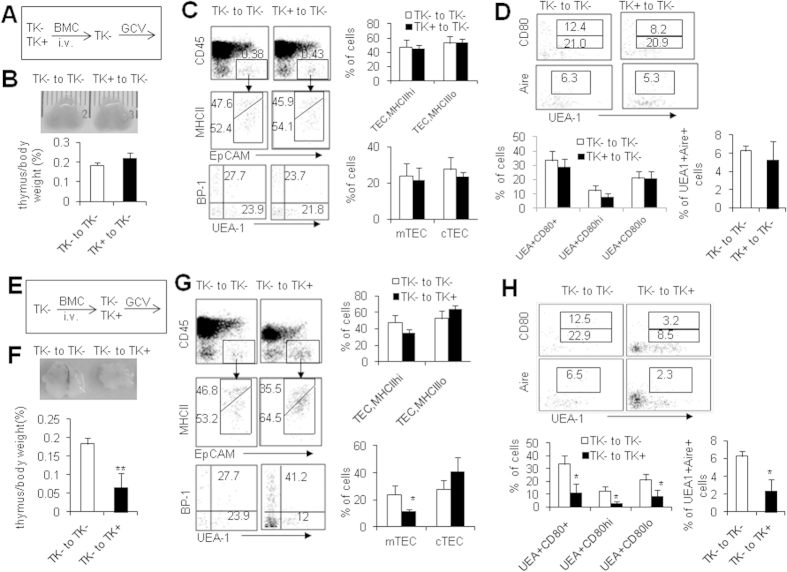
Essential roles of non-hematopoietic FSP1^+^ cells in TEC maintenance. (**A**) Full chimeric mice were generated by transplanting either TK^−^ and TK^+^ bone marrow cells (BMCs) to lethally irradiated TK^−^ mice. By 8 weeks after transplantation of BMCs, recipient mice were treated with GCV for 18 days, and the TEC subsets were assayed. (**B**) Representative photographs of thymus organs and the ratio of thymus weight to body weight in TK^−^ recipient mice received TK^−^ and TK^+^ BMCs. (**C**) Representative FACS staining and the frequencies of TECs, MHCII^high^ and MHCII^low^ TECs, mTECs and cTECs in TK^−^ recipient mice received TK^−^ and TK^+^ BMCs. (**D**) Representative FACS staining and the frequencies of CD80^high^, CD80^low^ and Aire expression in mTECs of TK^−^ recipient mice received TK^−^ and TK^+^ BMCs. (**E**) Full chimeric mice were generated by transplanting TK^−^ BMCs to lethally irradiated either TK^−^ and TK^+^ mice. By 8 weeks after transplantation of BMCs, recipient mice were treated with GCV for 18 days, and the TEC subsets were assayed. (**F**) Representative photographs of thymus organs and the ratio of thymus weight to body weight in TK^−^ and TK^+^ recipient mice received TK^−^ BMCs. (**G**) Representative FACS staining and the frequencies of TECs, MHCII^high^ and MHCII^low^ TECs, mTECs and cTECs in TK^−^ and TK^+^ recipient mice. (**H**) Representative FACS staining and the frequencies of CD80^high^, CD80^low^ and Aire expression in mTECs of TK^−^ and TK^+^ recipient mice were shown. Data presented are mean ± SD (N = 5). Representative results are shown from one of three independent experiments performed. *P < 0.05, **P < 0.01, ***P < 0.001 compared with TK^−^ mice.

Others and our studies (data not shown) showed that some subsets of immune cells like macrophages also expressed FSP1 in certain situations^30^,^38^, so it is essential to identify which cell type of thymic FSP1^+^ cells offered the supporting effects on mTEC homeostasis. We employed full bone marrow chimeric mouse models to elucidate whether FSP1^+^ immune cells participated in the impaired mTEC maintenance in GCV-treated FSP1-TK mice. First, we adoptively transferred either TK^−^ or TK^+^ bone marrow cells (BMCs) into lethally irradiated TK^−^ mice to establish the full chimeras (Fig. 3A)^39^. After 8 weeks after transplantation, recipient mice were treated with GCV for 18 days and the thymocyte and TEC subsets were assayed. In TK^−^ mice received TK^+^ BMCs, CD45^+^FSP1^+^ cells were from donors and would be deleted by GCV treatment, while thymic CD45^−^FSP1^+^ cells, which were from recipient mice, would not be deleted by GCV treatment. The identical thymus size, the ratio of thymus weight to body weight, total cell number, percentages of thymocyte, TECs, mTECs, cTECs and mature mTECs were observed in recipients receiving TK^−^ and TK^+^ BMCs respectively (Fig. 3B–D, and data not shown), indicating that ablation of hematopoietic-derived FSP1^+^ cells, did not impact TECs in this model. Reversely, another group of full chimeric mice were generated by transplanting TK^−^ BMCs into lethally irradiated either TK^−^ or TK^+^ mice (Fig. 3E). In this model, only non-hematopoietic FSP1^+^ cells in TK^+^ recipients received TK^−^ BMCs could be deleted after GCV treatment. Notably, smaller thymus size and lower ratio of thymus weight to body weight (P < 0.01, Fig. 3F), and altered total cell number and thymocytes in TK^+^ recipients of TK^−^ BMCs after GCV treatment compared with TK^−^ recipients of TK^−^ BMCs (data not shown). Furthermore, the percentages of mTECs and MHCII^high^ mature TECs but not cTECs were significantly decreased in GCV-treated TK^+^ recipients (P < 0.05, Fig. 3G). Moreover, the percentages and cell number of mature EpCAM^+^UEA-1^+^ mTECs including CD80^high^, CD80^+^ and Aire^+^ cells were markedly decreased (P < 0.05, Fig. 3H and data not shown). Based on all these data, we concluded that non-hematopoietic FSP1^+^ cells, which were proven to be one of thymic fibroblast subpopulations, played an important role in maintaining TEC particularly mTEC homeostasis in mice.

### Thymic FSP1^+^ fibroblasts promote mTEC regeneration

**Figure 4:**
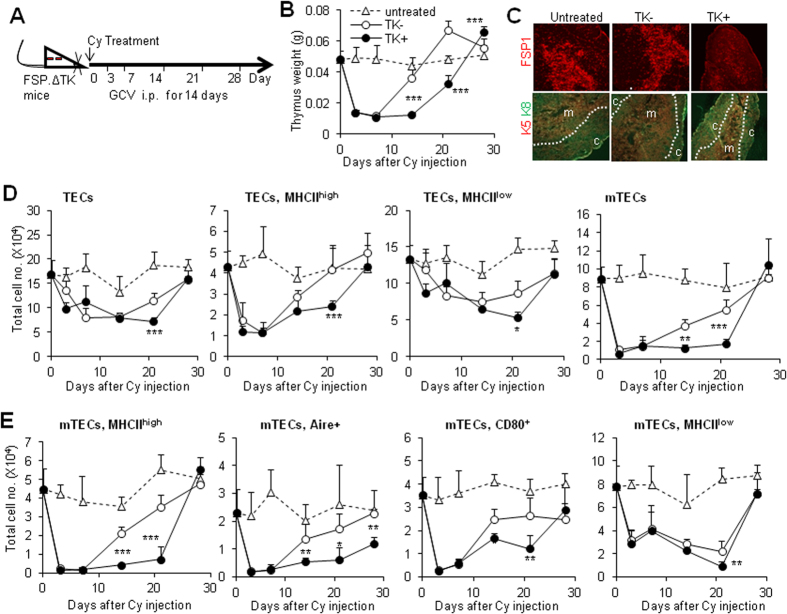
Deletion of FSP1^+^ cells significantly delayed thymus recovery. (**A**) Cyclophosphamide (Cy)-induced thymus regeneration model was established in TK^−^ and TK^+^ mice. These mice were treated with GCV for 14 days during thymus recovery. TECs subsets were analyzed at indicated time points. (**B**) The recovery curve of thymus weight of untreated (triangle), TK^−^ (open cycle) and TK^+^ (closed cycle) mice at various time-points after Cy injection. (**C**) Thymic sections from untreated and Cy/GCV-treated TK^−^ and TK^+^ mice after 14 days recovery were stained for the expression of FSP1 (the upper panel) and CK5/CK8 (the lower panel). (**D**) Recovery of cell number of TECs, TEC^high^, TEC^low^ and mTECs from Cy/GCV-treated TK^−^ and TK^+^ mice at different time points. (**E**) The recovery of mTECs of MHCII^high^, Aire^+^, CD80^+^ and MHCII^low^ in Cy/GCV-treated TK^−^ and TK^+^ mice at indicated time points were present. Data presented are the mean ± SD (N = 6), representing one representative of three independent experiments with identical results. *P < 0.05, **P < 0.01 and ***P < 0.001 compared with TK^−^ controls.

To assess the roles of thymic FSP1^+^ fibroblasts in thymic regeneration, we applied thymus regeneration model to detect the recovery efficiency of TEC subsets in the FSP1^+^ cell-deleted mouse model. Immunosuppressive agent cyclophosphamide (Cy) was used to induce thymus atrophy in TK^−^ and TK^+^ mice as reported previously^40^. GCV was injected for 14 days to delete proliferating FSP1^+^ cells during thymus recovery, thymic cellular composition and phenotype were then detected at indicated time points (Fig. 4A). In WT mice, Cy treatment induced severe thymus damage within 3 days, then recovery commenced, reaching almost normal levels by day 14 (Fig. 4B and suppl. Fig. 3A). However, deletion of FSP1^+^ cells by GCV in TK^+^ mice (Fig. 4C, the upper panel) caused significantly delayed thymus regeneration, as indicated by the observation showing that their thymus weight was particularly lower than those in TK^−^ mice at 14 and 21 days, although they could eventually recover to normal level at 28 days (Fig. 4B and suppl. Fig. 3A). Immunofluorescence analysis of thymus section from 14 days after Cy and GCV treatment stained with CK5 and CK8 revealed a significant decrease in medullary area in TK^+^ mice than TK^−^ mice (Fig. 4C, the lower panel). Meanwhile, the recovery ratio of the thymus weight to body weight, total thymocyte number and cell number of thymocyte subsets in TK^+^ mice were remarkably delayed (suppl. Fig. 3B–D). Importantly, the CD45^−^EpCAM^+^ TECs including MHCII^high^ and MHCII^low^ subsets in TK^+^ mice recovered in a slower manner than control mice (Fig. 4D). mTECs including relatively mature ones like MHCII^high^, CD80^+^ and Aire^+^ cells were unable to be recovered efficiently in TK^+^ mice as control mice after Cy and GCV treatment (Fig. 4D,E). Among them, Aire^+^ mTECs in TK^+^ mice could not recovered to normal level even 28 days after treatment (Fig. 4E). In contrast to mTECs, the total cell number of cTEC including MHCII^high^ and MHCII^low^ cells in TK^+^ mice were similar as in TK^−^ mice (suppl. Fig. 3E–G). Thus, these results demonstrated that FSP1^+^ cells were essential for mTEC regenerative potentiality after thymus injury.

Furthermore, we applied full bone marrow chimera to address whether hematopoietic or non-hematopoietic FSP1^+^ cells play the critical role in the thymus regeneration. 8 weeks after transplantation of either TK^−^ or TK^+^ BMCs into lethally irradiated TK^−^ mice, recipient mice were treated with Cy and GCV as same as thymus regeneration model (suppl. Fig. 4A) and analyzed 14 days after Cy treatment. Identical ratio of the thymus weight to body weight, total cell number, the percentage of thymocytes, TECs, mTECs and mature mTECs were observed in recipients receiving both TK^−^ and TK^+^ BMCs (suppl. Fig. 4B–F), suggesting that hematopoietic-derived FSP1^+^ cells did not impact thymic regenerative capacity. However, in full chimeric mice generated by transplanting TK^−^ BMCs to either TK^−^ or TK^+^ mice (suppl. Fig. 4G), we discovered significantly decreased ratio of thymus weight to body weight and total cell number in TK^+^ recipients of TK^−^ BMCs in which only CD45^−^FSP1^+^ cells were deleted (suppl. Fig. 4H). Notably, the percentage of CD4^+^CD8^+^ thymocytes, and MHCII^high^, CD80^+^ and Aire^+^ mature mTECs were also significantly decreased in TK^+^ recipients (suppl. Fig. 4I–L). These results indicated that thymic FSP1^+^ fibroblasts are important for regulating mTEC regenerative potentiality.

### Thymic FSP1^+^ cells control TEC proliferation

**Figure 5:**
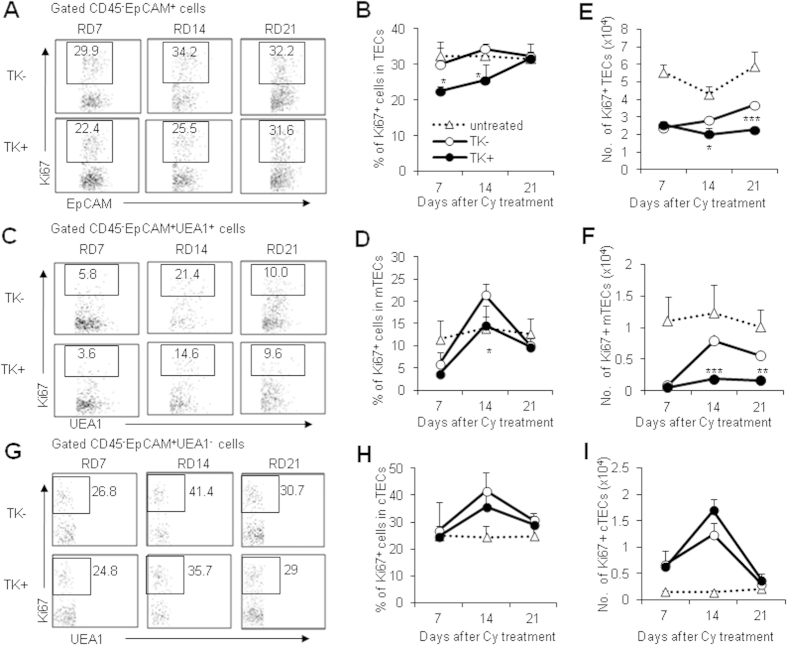
Deletion of FSP1^+^ cells impacted TEC proliferation. (**A**) Representative FACS profiles for Ki67 staining in the gated thymic CD45^−^EpCAM^+^ cells of Cy/GCV-treated TK^−^ and TK^+^ mice at various time points. (**B**) The percentages of Ki67^+^ cells in CD45^−^EpCAM^+^ TECs in untreated as well as Cy/GCV-treated TK^−^ and TK^+^ mice. (**C**) Dot plots of UEA-1 *vs* Ki67 expression on CD45^−^EpCAM^+^UEA-1^+^ TECs at different time points. (**D**) The percentages of Ki67^+^ cells in mTECs in Cy/GCV-treated TK^−^ and TK^+^ mice were shown. The cell number of CD45^−^EpCAM^+^Ki67^+^ TECs (**E**) and the cell number of Ki67^+^ mTECs (**F**) in Cy/GCV-treated TK^−^ and TK^+^ mice was summarized. (**G**) Dot plots of UEA-1 *vs* Ki67 expression on CD45^−^EpCAM^+^UEA-1^−^ TECs at different time points. (**H**) The percentages of Ki67^+^ cells in cTECs in Cy/GCV-treated TK^−^ and TK^+^ mice were shown. (**I)** The cell number of Ki67^+^ cTECs in Cy/GCV-treated TK^−^ and TK^+^ mice was summarized. Data represent the mean ± SD (n = 4 mice/group) from one of four independent experiments. **P < 0.01 and ***P < 0.001 (TK^−^ *vs* TK^+^).

Cy-induced thymic atrophy involved dramatic apoptosis of thymocytes and TECs, thus thymic regeneration depends on constant cell proliferation. Within thymus regeneration model, we postulated that defects in cell proliferation might contribute to the delayed recovery of TECs and mTECs in FSP1^+^ cell deleted mice. To address this possibility, we detected the proliferative marker Ki67 expression in TECs and mTECs at different time point after Cy and GCV treatment to investigate the cell proliferation rate of TECs and mTECs. According to the dynamics of thymus regeneration, we detected their proliferative capacity at 7, 14 and 21 days after Cy treatment. The percentage of Ki67^+^ cells was significantly lower in TECs and UEA-1^+^ mTECs of TK^+^ mice compared with the controls (Fig. 5A–D). Moreover, the absolute cell number of Ki67^+^ TECs and mTECs in TK^+^ mice were significantly lower than those in TK^−^ mice after Cy and GCV treatment during recovery (Fig. 5E,F). However, the percentages and cell numbers of Ki67^+^cTECs were similar in TK^−^ and TK^+^ mice after Cy and GCV treatment during recovery (Fig. 5G–I). Thus, deletion of FSP1^+^ fibroblasts impaired the cell proliferation of mTECs after injury, which might contribute to the defects of thymic regeneration in FSP1^+^ cells-deleted mice.

### Thymic FSP1^+^ fibroblasts promote TEC proliferation via IL-6 and FGF7

**Figure 6:**
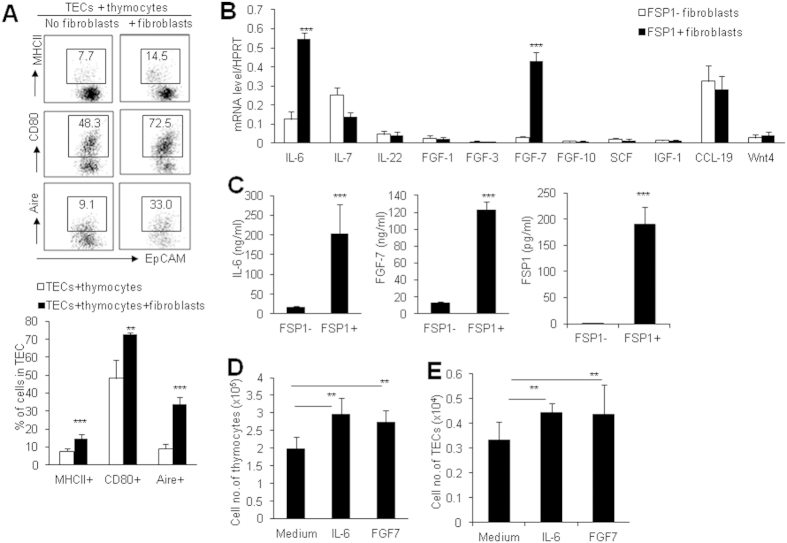
Thymic FSP-1^+^ fibroblasts promoted TEC proliferation by IL-6 and FGF7. RTOC were established by re-aggregating culture of TECs and thymocytes with or without thymic FSP1^+^ fibroblasts. (**A**) Representative FACS profiles and the frequencies of MHCII, CD80 and Aire expression in the gated CD45^−^EpCAM^+^ cells after RTOC culture with or without thymic FSP1^+^ fibroblasts. (**B**) The expression of cytokines in thymic FSP1^−^ and FSP1^+^ fibroblasts were determined by Real-time PCR. Data are representative of 2–3 independent experiments (3 samples each group per time). (**C**) The levels of IL-6, FGF7 and FSP1 in cell culture supernatants of primary thymic FSP1^−^ and FSP1^+^ fibroblasts were detected by ELISA. (**D**) The total cell numbers of thymic lobes cultured with IL-6 (100 ng/ml) and FGF7 (100 ng/ml) for 6 days in FTOC were summarized. (**E**) The total cell numbers of CD45^−^EpCAM^+^ TECs in thymic lobes cultured with IL-6 (100 ng/ml) and FGF7 (100 ng/ml) for 6 days in FTOC were summarized. Representative results are shown from one of three independent experiments performed. Data presented are mean ± SD (N = 6). *P < 0.05, **P < 0.01 and ***P < 0.001 (FSP1^−^ *vs* FSP1^+^ or between the indicated groups).

In order to address the potential mechanisms involved in the regulatory roles of thymic FSP1^+^ fibroblasts in maintaining thymic microenvironment and promoting thymus regeneration, we used the *in vitro* cultured thymic fibroblasts in re-aggregated thymic organ culture (RTOC) system. In RTOC, murine TECs and thymocytes were aggregated with or without mitomycin C-treated thymic FSP1^+^ fibroblasts. After 5 days culture, the results showed that thymic FSP1^+^ fibroblasts could remarkably enhance MHCII, CD80 and Aire expression on TECs (Fig. 6A), suggesting that thymic FSP1^+^ fibroblasts could promote TEC maturation.

Thymic fibroblasts are usually considered as nutritious cells to promote thymocyte and TEC proliferation by providing immune modulators. To identify the molecular mechanisms that FSP1^+^ fibroblasts regulate TEC maintenance and regeneration, we examined the expression profile of a series of cytokines which are particularly critical for TEC proliferation in thymic FSP1^−^ and FSP1^+^ fibroblasts^16^,^41^,^42^. Real-time PCR results revealed that, among the detected molecules, strikingly high signal for IL-6 and FGF7 were obtained in FSP1^+^ fibroblasts compared to FSP1^−^ group (P < 0.001, Fig. 6B). These results were further confirmed by ELISA assays detecting cell culture supernatants from FSP1^−^ and FSP1^+^ fibroblasts (P < 0.001, Fig. 6C). IL-6 and FGF7 have been demonstrated to be very important growth factors for TEC proliferation *in vitro* and *in vivo*^16^,^43^,^44^. When we performed fetal thymus organ culture (FTOC), addition of IL-6 and FGF7 significantly increased total cell number and TEC cell number in the cultured thymic lobes respectively (Fig. 6D,E), confirming that IL-6 and FGF7 markedly supported TEC proliferation^44^.

Apart from locating in the nucleus and cytoplasm, FSP1 was recently found to be released to extracellular space^45^,^46^. By interacting with their cell surface receptors, FSP1 possesses a wide range of biological functions, such as regulation of cell survival and proliferation^45^. To determine whether FSP1 could be secreted by FSP1^+^ fibroblasts, we also detected FSP1 protein levels in cell culture supernatants by ELISA. Clearly, thymic FSP1^+^ fibroblasts could secrete FSP1 protein into the culture medium (P < 0.001, Fig. 6C), leading us to examine the contribution of FSP1 protein in TEC proliferation and function.

### Thymic FSP1^+^ fibroblasts enhance TEC maturation via FSP1

**Figure 7:**
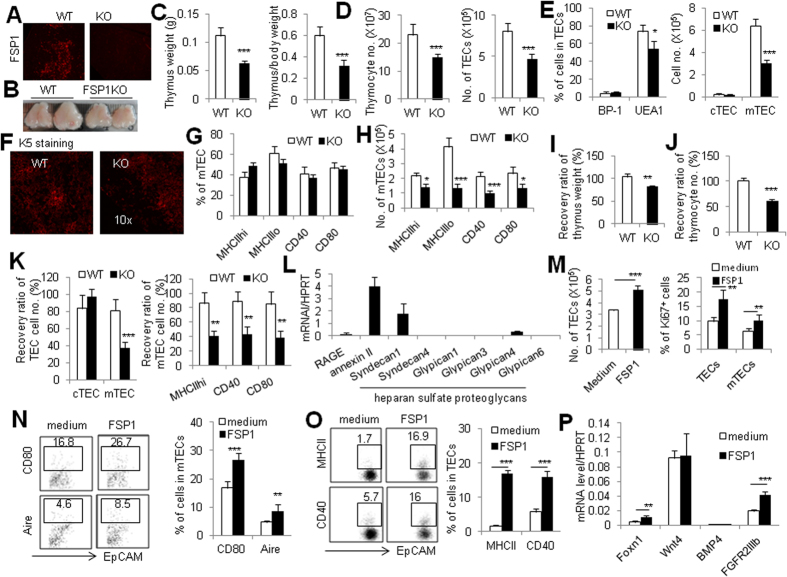
FSP1 directly regulates TEC proliferation and differentiation. (**A**) Frozen thymic section from 6–8wk WT and FSP1KO mice were stained with FSP1. (**B**) Representative photographs of thymus organs in WT and FSP1KO mice was shown. (**C**) Thymus weight and the ratio of thymus weight to body weight in FSP1KO mice were significantly lower than in WT mice. (**D**) Total cell numbers of thymocyte and TECs in WT and FSP1KO mice were presented. (**E**) The percentage and the total cell number of cTECs and mTECs in WT and FSP1KO mice. (**F**) Frozen thymic sections from WT and FSP1KO mice were stained with CK5, revealing decreased thymic medullary area in FSP1KO mice. The frequencies (**G**) and the total cell number (**H**) of MHCII^high^, CD40^+^, CD80^+^ mTECs in WT and FSP1KO mice were summarized. (**I**) The recovery ratio of thymus weight in FSP1KO mice was lower than in WT mice after Cy-treatment. (**J**) The recovery ratio of total cell number of thymocytes in WT and FSP1KO mice after Cy-treatment were shown. (**K**) The recovery ratio of cTECs and mTECs, and mTECs expressing MHCII^high^, CD40^+^ and CD80^+^ in WT and FSP1KO mice were summarized. Data is mean ± SD (4–6 mice/group) from one of two independent experiments. (**L**) The mRNA levels of RAGE, annexin II and heparan sulfate proteoglycans in cultured primary TECs and thymocytes were assayed by Real-time PCR. (**M**) FSP1 significantly increased the total cell number of TECs and the percentage of Ki67^+^ cells in TECs and mTECs after the fetal thymus was cultured with FSP1 in FTOC system for 6 days were summarized. (**N**) Representative FACS and the percentage of CD80 and Aire expression on mTECs were significantly increased after thymi were cultured in FTOC system with FSP1 for 6 days. (**O**) Representative FACS and the percentage of MHCII and CD40 expression on TECs were significantly increased after primary TECs were cultured with FSP1 for 5 days. (**P**) The expression of Foxn1, Wnt4, BMP4 and FGFR2IIIb in TECs cultured with or without FSP1 were detected by Real-time PCR. Data is mean ± SD (3–5 sample/group) from one of two independent experiments. *P < 0.05, **P < 0.01, and ***P < 0.001 (FSP1KO *vs* WT or between the indicated groups).

To better comprehend the effect of FSP1 protein in TEC development and function. We investigated the thymus and TEC phenotype of FSP1KO mice. These FSP1KO mice were fertile and displayed no gross abnormalities within 3 months after birth. Immunofluorescence analysis of thymic sections from WT and FSP1KO mice revealed that FSP1 expression was eliminated clearly (Fig. 7A). Moreover, FSP1KO mice had a thymus defect as evidenced by the smaller thymus size, lower thymus weight and lower ratio of thymus weight to body weight (P < 0.001, Fig. 7B,C) compared with age-matched WT mice. The total cell number of thymocytes and TECs were significantly decreased in FSP1KO mice (P < 0.001, Fig. 7D). The percentage and cell number of mTECs but not cTECs were remarkably decreased in FSP1KO mice than those in WT mice (P < 0.05, Fig. 7E). Consistently, FSP1KO mice had significantly smaller medulla compared with WT mice, as shown in the thymic sections stained with CK5 (Fig. 7F). The cell number of mTECs including MHCII^high^, CD40^+^ and CD80^+^ cells were markedly decreased in FSP1KO mice, though their percentage were unchanged (Fig. 7G,H). These observations suggested that FSP1 played an important role in maintaining mTEC compartment and enhancing thymic size and cellularity.

More impressively, we found that FSP1 was involved in thymic epithelium regeneration after thymus damage. We detected the recovery efficiency of TEC subsets in FSP1KO and WT mice treated with Cy. Because FSP1KO mice had smaller thymus and lower cellularity than WT mice before Cy treatment, we thus utilized the recovery ratio of cell number to determine the thymic regeneration ability in these assays. By 14 days after Cy treatment, the recovery ratio of the thymus weight, total cell number and thymocyte subsets in FSP1KO mice were strikingly lower than WT mice (P < 0.001, Fig. 7I,J). Notably, mTECs, but not cTECs, and mature mTECs including MHCII^high^, CD40^+^ and CD80^+^ cells in FSP1KO mice were also unable to be recovered efficiently as WT mice (P < 0.001, Fig. 7K). These data indicated that FSP1 deficiency significantly affected the regenerative potentiality of mature mTECs after thymus damage.

It has been reported that FSP1 could interact with cell surface receptors such as receptor for advanced glycation end products (RAGE), annexin II, and heparan sulfate proteoglycans^45^. To address the possibility that FSP1 directly regulates TEC differentiation, we first investigated the expression of cell surface receptors for FSP1 in TECs. Real-time PCR assays of the cultured TECs revealed that several FSP1 receptors were expressed in TECs, among which annexin II and syndecan 1, one of heparan sulfate proteoglycans, expressed relatively high (Fig. 7L). We next assessed the potential role of FSP1 protein involved in regulating TECs’ differentiation. Importantly, addition of purified FSP1 protein significantly increased the total cell number of TECs, the percentage of Ki67^+^ cells in TECs and mTECs, as well as the expression of the mature markers CD80 and Aire on mTECs in FTOC culture system (Fig. 7M,N), indicating the supporting role of FSP1 on mTEC proliferation and maturation. Consistently, in the *in vitro* TEC culture system, FSP1 protein could significantly increase MHCII and CD40 expression on TECs (Fig. 7O). Meanwhile, FSP1 treatment significantly increased the key TEC regulator Foxn1 expression in TECs as detected by Real-time PCR assay (Fig. 7P), which might contribute to the enhancement of MHCII and CD40 expression on TECs. Additionally, FSP1 also increased the expression of FGFR2IIIb, the receptor for FGFs^17^ in TECs (Fig. 7P), indicating that FSP1 could promote the ability of TECs utilizing FGFs. Therefore, these data illustrated that thymic FSP1^+^ fibroblasts could enhance TEC proliferation and differentiation via directly producing FSP1 protein.

## Discussion

As the primary lymphoid organ, thymus is the place for T cell development and maturation, playing a crucial role in establishment of T cell immunity and self-tolerance. Thymus possesses a complex microenvironments consisting of many cell components in which TECs and thymocytes are the most abundant and important compositions. Besides, fibroblasts are also an irreplaceable stromal cell type in thymic microenvironments by supporting thymic structure and function^1^. However, due to the heterogeneity and lack of specific markers^25^,^47^,^48^, the identification, development and function of thymic fibroblasts remain poorly understood. In our present study, we revealed that non-hematopoietic CD45^−^FSP1^+^ cells in the thymus represent a subpopulation of fibroblasts as supported by the following evidences and characteristics: 1) The cultured primary thymic CD45^−^FSP1^+^ cells displayed classical spindle shape and express fibroblast marker vimentin^49^, but not other cell type markers like cytokeratin, CD11b, CD11c and UEA-1. 2) Thymic CD45^−^FSP1^−^ fibroblasts were smaller but CD45^−^FSP1^+^ cells were larger. 3) In the primary cell culture, thymic CD45^−^FSP1^−^ fibroblasts underwent transformation to CD45^−^FSP1^+^ cells, but no CD45^−^FSP1^+^ cells turn into CD45^−^FSP1^−^ cells. This observation also indicates that thymic CD45^−^FSP1^+^ fibroblasts likely represent a more mature state than CD45^−^FSP1^−^ fibroblasts. 4) Thymic CD45^−^FSP1^+^ fibroblasts produced significantly more IL-6 and FGF7 than CD45^−^FSP1^−^ cells. 5) Little co-location of FSP1 with cytokeratin, CD31 and α-SMA in the thymus assayed by immunohistochemical staining and FSP1-GFP reporter assays implied that thymic CD45^−^FSP1^+^ cells are not epithelium, endothelium or perivascular smooth muscle cells. 6) Immunofluorescence staining of the thymus showed that only a fraction of FSP1^+^ cells were co-localized with MTS15 and ER-TR7, respectively. Thymic FSP1^+^ fibroblasts extensively exist in the thymic medullary area, which is different from the distribution MTS15^+^ and ER-TR7^+^ fibroblasts. In addition, the majority of CD45^−^FSP^+^ fibroblasts express mesenchymal cell markers BP-1, gp38, PDGFRα and PDGFRβ^47^,^50^,^51^, indicating that thymic CD45^−^FSP1^+^ fibroblasts were derived from mesenchymal precursors. The previous view of the origin for thymic fibroblast holds that they are developed from neural crest (NC)-derived mesenchyme^52^,^53^. In a recent study, Komada *et al.* using double-transgenic mice defined that thymic mesenchymal PDGFRα and PDGFRβ expressing cells were composed of both NC and mesoderm-derived cells, contributing to perivascular cells and endothelial cells, respectively^54^. However, our study showed that thymic CD45^−^FSP^+^ fibroblasts express PDGFRα and PDGFRβ but not α-SMA and CD31. Thus, the precise origins of these FSP1^+^ fibroblasts are still need future investigation^52^,^53^. All these findings shed lights on the heterogeneity of thymic fibroblasts.

Thymic fibroblasts were considered to play a role in supporting thymus structure by distribution at thymic subcapsule, septae, and near vasculature, and also promoting thymic cellularity by producing secretory mediators^16^. However, few studies directly investigated the function of fibroblasts themselves in the thymus, but indirectly studied their mesenchymal precursors^19^,^42^ or cytokines executors such as FGFs^44^,^53^,^55^. Our research using FSP1^+^ cells-deleted mice (FSP1-TK), full bone marrow chimera mice, combined with thymus regeneration models provide evidence on the function of thymic FSP1^+^ fibroblasts. We demonstrated that CD45^−^FSP1^+^ cells played an essential role in thymus maintenance as verified by small thymus size, decreased thymus weight and total cell number after systemic ablation of FSP1^+^ cells. Importantly, FSP1^+^ cells were required for mTEC homeostasis because of decreased mature mTECs including MHCII^high^, CD80^+^, Aire^+^ cells in FSP1^+^ cells-deleted thymus. Moreover, deletion of FSP1^+^ cells significantly impaired thymus full recovery after Cy-induced injury. During the course of thymus regeneration, we found the recovery of thymus weight, thymocytes and TECs, particularly mature mTECs, was remarkably delayed in FSP1^+^ cells-deleted mice. To avoid the effect of hematopoietic FSP1^+^ cells, we performed studies using full bone marrow chimeras and concluded that non-hematopoietic CD45^−^FSP1^+^ cells which were proven to be fibroblasts population played the critical role in the thymus maintenance and regeneration. We failed to observe the detectable impacts of hematopoietic FSP1^+^ cells on TEC maintenance and regeneration in physiological and pathological situations.

Deletion of thymic FSP1^+^ fibroblasts significantly impacted mTEC proliferation as supported by the decreased Ki67 expression during thymic regeneration. Since many cytokine mediators, which were produced mostly by thymic fibroblasts, were involved in TEC proliferation^16^,^43^,^44^, Real-time PCR and ELISA results verified that thymic FSP1^+^ fibroblasts produced high level of IL-6 and FGF7 than thymic FSP1^−^ fibroblasts. IL-6 and FGF7 have been demonstrated to be very important growth factors for TEC proliferation *in vitro* and *in vivo*^16^,^43^,^44^. Our data showed that exposure to IL-6 and FGF7 significantly increased total cell number and TEC cell number of the cultured thymic lobes in FTOC culture, confirming that IL-6 and FGF7 markedly supported TEC proliferation^55^. Therefore, thymic FSP1^+^ fibroblasts control TEC maintenance and regeneration through their ability to produce large amount of IL-6 and FGF7.

FSP1, known as S100A4, belonging to S100 family has been extensively investigated in tumorigenesis. A wealth of information illustrated that FSP1 promoted cancer progression by enhancing cell proliferation, motility, invasiveness, metastasis and angiogenesis^45^,^56^,^57^,^58^. Except for location in nucleus and cytoplasm, FSP1 was revealed to be secreted into extracellular space to exert their effects by interacting with the cell surface receptors^45^,^46^,^59^. Our results showed that primary thymic FSP1^+^ fibroblasts could release FSP1 into the culture medium, offering the possibility that thymic FSP1^+^ fibroblasts might regulate TECs through the released FSP1 protein. Three types of receptors, RAGE, annexin II and heparan sulfate proteoglycans were found to be surface receptors for FSP1^45^. We found that TECs expressed FSP1 receptors mainly including annexin II and syndecan1, further indicating that FSP1 could directly regulate TEC function. It is true that FSP1 supports TEC proliferation and maturation as evidenced by the *in vitro* and in *vivo* studies. FSP1KO mice had small thymus, low cellularity, decreased mTECs including CD40^+^ and CD80^+^ mTECs compared with WT mice and displayed inefficient regeneration after thymus damage. Addition of FSP1 protein significantly increased the cell number of TECs and mTECs, as well as the expression of CD80, CD40 and Aire expression on mTECs in FTOC and TEC culture assays. Furthermore, FSP1 significantly increased the expression of the key transcription factor for TECs, Foxn1^60^,^61^, and one of the important nursing receptors for TECs, FGFR2IIIb^53^, in mouse TEC culture system. Thus, FSP1 itself acted as a key direct regulator in TEC development through up-regulation of Foxn1 and FGFR2IIIb.

Taken together, our studies reveal a unique subpopulation of thymic fibroblasts expressing FSP1, which was mainly located in the thymic medullary zone. Thymic FSP1^+^ fibroblast subset plays an essential role in thymic medullary maintenance and regeneration under physiological and pathological situations. Thymic FSP1^+^ fibroblasts regulate the proliferation and differentiation of mTECs through providing IL-6, FGF7 and FSP1.

## Materials and Methods

### Mice

C57BL/6 and BALB/c mice were purchased from Beijing University Experimental Animal Center (Beijing, China). FSP1-TK transgenic mice, in which proliferating FSP1^+^ cells can be depleted selectively upon administration of ganciclovir^35^,^36^, were obtained from Dr. Eric G. Neilson (Northwestern University, Feinberg School of Medicine). FSP1-GFP reporter mice^33^,^62^ and FSP1KO mice^30^ were purchased from Jackson laboratory. FSP1-GFP and FSP1KO mice have a C57BL/6 genetic background. FSP1-TK mice have a BALB/c genetic background. All mice were bred and maintained in specific pathogen-free conditions. Six- to 8-week-old sex- and age-matched mice or littermates were used for experiments. All animal experiments were performed in accordance with the approval of the Animal Ethics Committee of the Institute of Zoology, Beijing, China.

### Antibodies and flow cytometry

The following biotinylated or fluorochrome-conjugated antibodies were used in flow cytometry detection. Biotinylated Ulex europaeus agglutinin (UEA-1) was obtained from Vector Laboratories and revealed with streptavidin-Phycoerythrin (PE) (BD Pharmingen), or Alexa Fluor® 610—R-Phycoerythrin (Invitrogen). The following antibodies were purchased from Biolegend: anti-CD45-PerCP/Cy5.5, (Clone 30-F11), anti-EpCAM-FITC, anti-EpCAM-PE (Clone G8.8) , Alexa Fluor® 488 anti-I-A/I-E (clone M5/114.15.2), anti-CD80-PE (clone 16-10A1), anti-CD40-PE (clone 3/23), anti-CD8-PE/CY5 (clone 53-6.7), anti-podoplanin/gp38-PE (clone 8.1.1), biotinylated anti-CD140b/PDGFRβ (clone APB5) and anti-CD31-PE (clone 390). The following antibodies were from eBioscience: anti-BP-1-PE (Ly51, clone 6C3) and biotinylated anti-CD140a/PDGFRα (clone APA5). Anti-CD4-FITC (clone RPA-T4) was from BD Pharmingen. Anti-MTS15 mAb is a gift of Prof. Richard Boyd (Monash University). Surface staining of cell suspensions was performed in PBS/0.1% BSA/0.02% NaN_3_ solution at 4˚C. Intracellular staining for Aire-FITC (kindly provided by Prof. Francois-Xavier HUBERT, Walter and Eliza Hall Institute of Medical Research) or Alexa Fluor® 647 (clone 5H12; eBioscience) and Ki-67 staining (BD Pharmingen) were performed using fixation buffer (eBioscience) and permeabilization buffer (eBioscience), according to the manufacturer’s protocol.

### Immunohistology and Immunofluorescence

For analysis of thymic medulla and cortex by immunohistology, thymi from GCV treated TK^−^ and TK^+^ mice were fixed in 4% formalin and embedded in paraffin blocks. Sections (5 μm) were stained with hematoxylin and eosin (H&E) and examined by light microscopy. For immunofluorescence, serial sections (5 μm) from OCT-embedded frozen tissues or primary cultured cells were fixed in cold acetone or 4% polyoxymethylene and blocked in PBS/1% BSA, washed in PBS/0.05% Tween and incubated with optimal dilutions of fluorochrome-conjugated antibodies: Alexa Fluor® 488 anti-I-A/I-E, anti-CD31-PE (Biolegend), anti-CD11c-PE, anti-CD11b-FTIC (BD Pharmingen), and anti-F4/80-PE (eBioscience), or with first Abs: anti-cytokeratin 5, anti-FSP1, anti-ER-TR7 (Abcam), anti-cytokeratin 8 (Tromal-1; Developmental Studies Hybridoma Bank), Biotinylated UEA-1, anti-vimentin (BD Pharmingen), anti-α-SMA, anti-Pan-CK (Sigma, Cat no. C5992), anti-CD140a/PDGFRα (R&D Systems) and anti-MTS15 Ab for 2 h at room temperature before washing and incubating with secondary reagents: Alexa Fluor® 546 Goat anti-Rabbit/mouse IgG (H+L), Alexa Fluor® 488 Goat anti-Rat IgG (H+L) (Invitrogen), Dylight 488 Goat anti-Rabbit/mouse IgG (ZSGB-Bio) and streptavidin-PE (BD PharMingen). Control slides were incubated with isotype-matched Ig. Images were acquired with a two-photon microscopy (Carl Zeiss, Inc.).

### Thymic stromal cell isolation and *in vitro* culture of TECs and thymic fibroblasts

Thymic stromal cells from the postnatal thymus were isolated as previously described^40^,^63^. In Brief, freshly dissected thymi were cut into 1-mm^3^ pieces, washed with DMEM medium with 2% FBS several times to remove the majority of thymocytes. The thymic fragments were then incubated at 37 °C for 10 min in 2 mL solution of 1 mg/mL collagenase D (equivalent to 0.1% w/v) with 20 U/mL DNAse I (both from Sigma). Enzymatic treatment was repeated 3 times (the final incubation with collagenase/dispase enzyme mixture) until all fragments were dispersed. Gentle agitation was performed periodically at mid- and end-points of each digestion. Cell suspensions from each digestion were pooled in PBS containing 1% FBS and 5 mM EDTA to neutralize digestion and remove cell aggregates. Cells were centrifuged, re-suspended and filtered to remove clumps. Phenotypes of TECs were analyzed by surface FACS staining.

For TEC cell culture, thymi from WT neonatal mice were digested as mentioned above. Small thymic fragments from each step were collected and pooled. Fragments were allowed to settle and washed twice with PCT medium (CnT07, CellnTEC). The remaining thymic explants were plated in 48-well plates with CnT07 medium and cultured at 37 °C and 5% CO_2_ for several days, during which TECs outgrew other stromal cells^40^. To determine the effect of FSP1 on TECs, cultured TECs were treated with 2 μg/ml FSP1 protein. After 5 days treatment, TECs were collected with trypsin (Sigma) digestion and analyzed for MHCII and CD40 expression by FACS.

For thymic fibroblast culture, thymi from FSP1-GFP neonatal mice were digested as mentioned above. Cell suspensions from each digestion were pooled, centrifuged and re-suspended in the DMEM medium with 10% FBS and cultured for several days during which fibroblasts outgrew other stromal cells. To inhibit cell proliferation, thymic fibroblasts were treated with 10 μg/ml mitomycin C for 4 hours at 37 °C and 5% CO_2_.

### FSP1^+^ cell deletion and thymus regeneration models

In systemic ablation model, FSP1-TK^+^ and control WT mice were injected i.p. with ganciclovir (GCV) at a dose of 20 mg/kg body weight twice a day for 18 consecutive days. FSP1-TK^+^, FSP1KO and control mice were injected i.p. with Cy at a dose of 100 mg/kg body weight per day for two consecutive days to induce thymus damage^40^. The first day after Cy treatment was considered as recovery day 1. For FSP1^+^ cells-deletion, TK^+^ and TK^−^ mice were treated with GCV two times every day for 14 days. Thymocytes and TECs of these mice were isolated at indicated time for analysis.

### Quantitative RT-PCR and ELISA

RNA was purified from cultured primary thymic fibroblasts and sorted TECs. mRNA was prepared using RNeasy Mini Kit (Qiagen) and the cDNA library was generated with Reverse Transcription System Kit (promega), according to the manufacturer’s protocol. qPCR was performed using SYBR Premix Ex Taq (Takara). Relative expression values for target genes normalized to HPRT were obtained. The primers used in the present study were listed in Table 1. To determine the protein level of IL-6, FGF7 and FSP1, cell culture supernatants from FSP1^−^ and FSP1^+^ fibroblasts were collected and measured separately with ELISA commercial kits according to the manufacturer’s protocol (USCN Life Science Inc.).

### Bone marrow chimeras

Bone marrow cells (BMCs) from 8-wk-old TK^−^ and TK^+^ mice were prepared, respectively. 1×10^7^ BMCs were injected into the tail vein of lethally irradiated 8-wk-old TK^−^ and TK^+^ recipients to set up full chimeras as described^40^,^64^. 8 wks after reconstitution, recipient mice were treated with GCV or with combination of Cy and GCV. Mice were sacrificed at the indicated time points, and thymocytes and TECs were isolated for analysis.

### Fetal thymus organ culture (FTOC) and re-aggregated thymic organ culture (RTOC) systems

FTOC was performed as described previously^65^. Briefly, thymic lobes were isolated from embryos 15.5 days postcoitus and were cultured for 5 days on the top of Nucleopore filters (Whatmann) placed in DMEM medium supplemented with 10% fetal bovine serum (FBS) (Gibco), 2 mM L -glutamine, 100 U/ml penicillin, 100 mg/ml streptomycin, and 50 mM 2-mercaptoethanol containing 1.35 mM 2’-deoxyguanosine (2-dGUO) (Sigma-Aldrich). To test the effect of IL-6, FGF7 and FSP1 using FTOC, 2-dGUO treated fetal thymic lobes were cultured in DMEM plus 10% FBS with recombinant IL-6 (100 ng/ml; Perprotech), recombinant FGF7 (100 ng/ml; R&D) or FSP1 (5 μg/ml). Six days after the stimulation, the lobes were harvested for flow cytometric analysis. For RTOC, *in vitro* cultured TECs and freshly isolated thymocytes were reaggregated with or without MitoC treated thymic fibroblasts at a ratio of 1:6:1 by centrifugation and the cell pellet of the aggregates were drawn in 2 μl into plastic tips to place onto the surface of nucleopore filters for 5 days of culture.

### Statistical analysis

All data are presented as the mean+SD. Student’s unpaired *t* test for comparison of means was used to compare groups. A *P* value less than 0.05 was considered to be statistically significant.

## Additional Information

**How to cite this article**: Sun, L. *et al.* FSP1^+^ fibroblast subpopulation is essential for the maintenance and regeneration of medullary thymic epithelial cells. *Sci. Rep.* **5**, 14871; doi: 10.1038/srep14871 (2015).

## Supplementary Material

Supplementary Information

## Figures and Tables

**Table 1:** The primers used in the present study.

Primers	Sense sequence	Anti-sense sequence
IL-6	5′-AACCGCTATGAAGTTCCTCTC -3′	5′-AATTAAGCCTCCGACTTGTGAA-3′
IL-7	5′-ATCCTTGTTCTGCTGCCTGTCA-3′	5′-ACCAGTGTTTGTGTGCCTTGTG-3′
IL-22	5′-TGTGCGATCTCTGATGGCTGTC-3′	5′-AGGTGCGGTTGACGATGTATGG-3′
FGF1	5′-TTCTTCAGTGCTGAGCCTACCA-3′	5′-CACGGTGCCATCAGGAAGGA-3′
FGF3	5′-CGCTACCAAGTACCACCTCCAG-3′	5′-CGAAGCATACAGCCGTCCTCTC-3′
FGF7	5′-AACGGCTACGAGTGTGAACT-3′	5′-CAACTGCCACGGTCCTGAT-3′
FGF10	5′-AGATGTCCGCTGGAGAAGG-3′	5′-AGTTGCTGTTGATGGCTTTGA-3′
SCF	5′-AGGAATGACAGCAGTAGCAGTA-3′	5′-CGTCCACAATTACACCTCTTGA-3′
IGF-1	5′-CGCTCTGCTTGCTCACCTTC-3′	5′-ACACTCATCCACAATGCCTGTC-3′
CCL-19	5′-TTCACGCCACAGGAGGACATCT-3′	5′-GGCAGCAGTCTTCCGCATCATT-3′
Wnt4	5′-CTC AAA GGC CTG ATC CAG AG-3′	5′-TCA CAG CCA CAC TTC TCC AG-3′
RAGE	5′-CAACTACCGAGTCCGAGTCTAC-3′	5′-GTCTCCTGGTCTCTTCCTTCAC-3′
annexin II	5′-GGACATTGCCTTCGCCTATCAG-3′	5′-TGGTTGGTTCGTGAGCAGATGA-3′
Syndecan1	5′-AGGATGGAACTGCCAATCAG-3′	5′-ATCCGGTACAGCATGAAAGC-3′
Syndecan4	5′-AACCACATCCCTGAGAATGC-3′	5′-AGGAAAACGGCAAAGAGGAT-3′
Glypican1	5′-CGACCGCTGCTGGAATGGAATT-3′	5′-GGAGCCACTGCCGTCATCACTA-3′
Glypican3	5′-TGTGCCCAAGGGTAAAGTTC-3′	5′-AGGTGGTGATCTCGTTGTCC-3′
Glypican4	5′-CGTTTGCAATGATGAGAGGA-3′	5′-GCCATGATCTGACGAAGGAT-3′
Glypican6	5′-CAACGAGGAGGAGTGCTGGAAC-3′	5′-GGTCATCACACGGAGAGCCATG-3′
Fonx1	5′-ACCTTGGGACTGACCTGGATG-3′	5′-CTGCCTCATTGCCTGTTTCTG-3′
BMP4	5′-ATCTGGTCTCCGTCCCTGATGG-3′	5′-CGTCGCTCCGAATGGCACTA-3′
FGFR2IIIb	5′-AGTCTGCCTGGCTCACTGTCCT-3′	5′- AGCTGGCTGGCTGCTGAAGTCT-3′
