# Supplementary material for: FSP1+ fibroblast subpopulation is essential for the maintenance and regeneration of medullary thymic epithelial cells
Source: Sci Rep. 2015 Oct 8;5:14871. doi: 10.1038/srep14871 (PMC4597222; doi:10.1038/srep14871)
Supplement: Supplementary Information [file srep14871-s1.pdf]

Supplemental Figures

FSP1^{+} fibroblast subpopulation is essential for the maintenance and regeneration of medullary thymic epithelial cells

Lina Sun[*,#], Chenming Sun[*,#], Zhanfeng Liang[*,#], Hongran Li[*], Lin Chen[†], Haiying Luo[*], Hongmei Zhang[*], Pengbo Ding[*], Xiaoning Sun[*], Zhihai Qin[†, §] and Yong Zhao [*, §]

[*] State Key Laboratory of Biomembrane and Membrane Biotechnology, Institute of Zoology, Chinese Academy of Sciences, Beijing, China; [†] Key Laboratory of Protein and Peptide Pharmaceuticals, Institute of Biophysics, Chinese Academy of Sciences, Beijing, China.

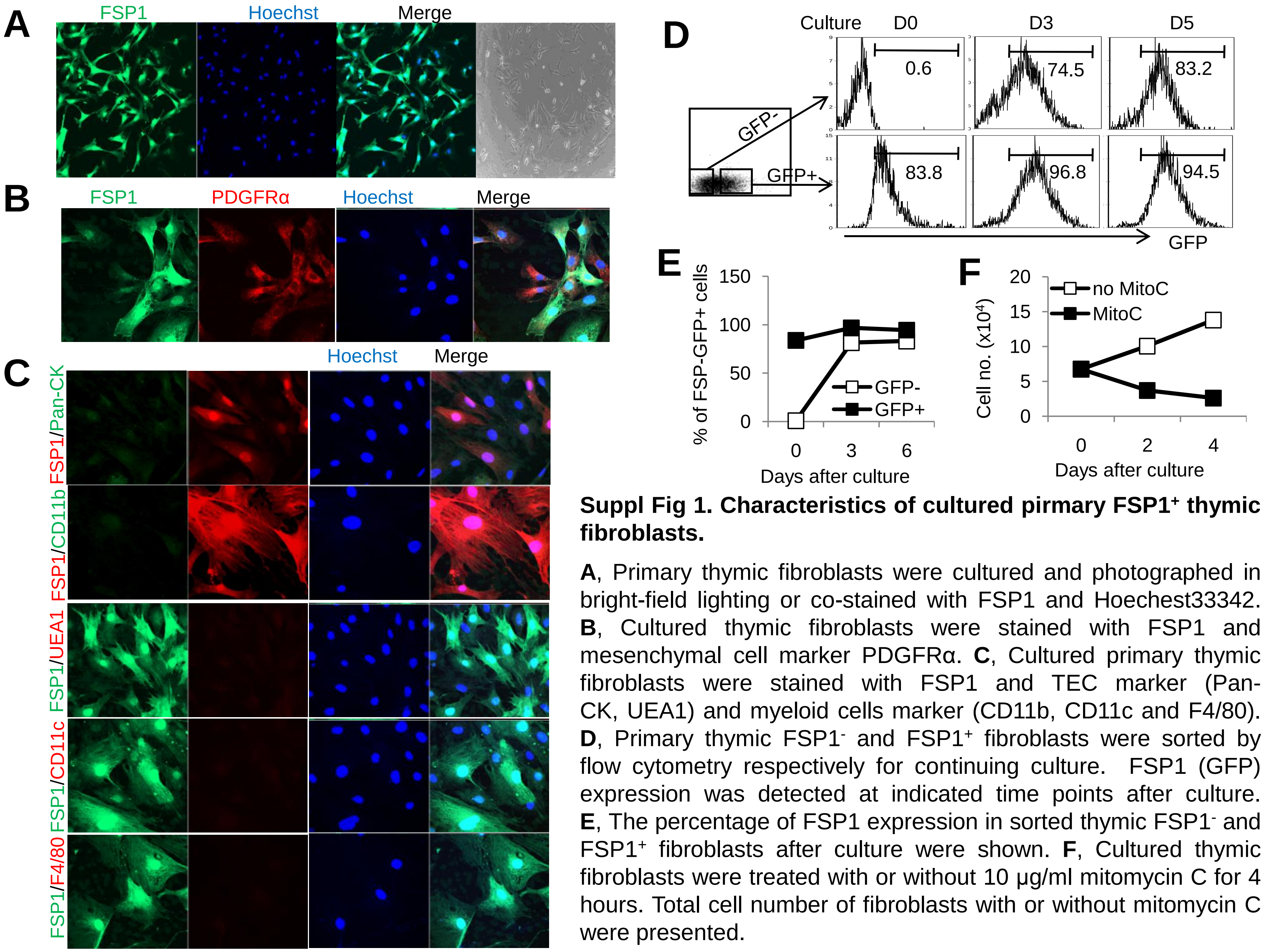

Suppl Fig 1. Characteristics of cultured pirmary FSP1^{+} thymic fibroblasts.

A, Primary thymic fibroblasts were cultured and photographed in bright-field lighting or co-stained with FSP1 and Hoechest33342. **B**, Cultured thymic fibroblasts were stained with FSP1 and mesenchymal cell marker PDGFRα. **C**, Cultured primary thymic fibroblasts were stained with FSP1 and TEC marker (Pan-CK, UEA1) and myeloid cells marker (CD11b, CD11c and F4/80). **D**, Primary thymic FSP1^{-} and FSP1^{+} fibroblasts were sorted by flow cytometry respectively for continuing culture. FSP1 (GFP) expression was detected at indicated time points after culture. **E**, The percentage of FSP1 expression in sorted thymic FSP1^{-} and FSP1^{+} fibroblasts after culture were shown. **F**, Cultured thymic fibroblasts were treated with or without 10 μg/ml mitomycin C for 4 hours. Total cell number of fibroblasts with or without mitomycin C were presented.

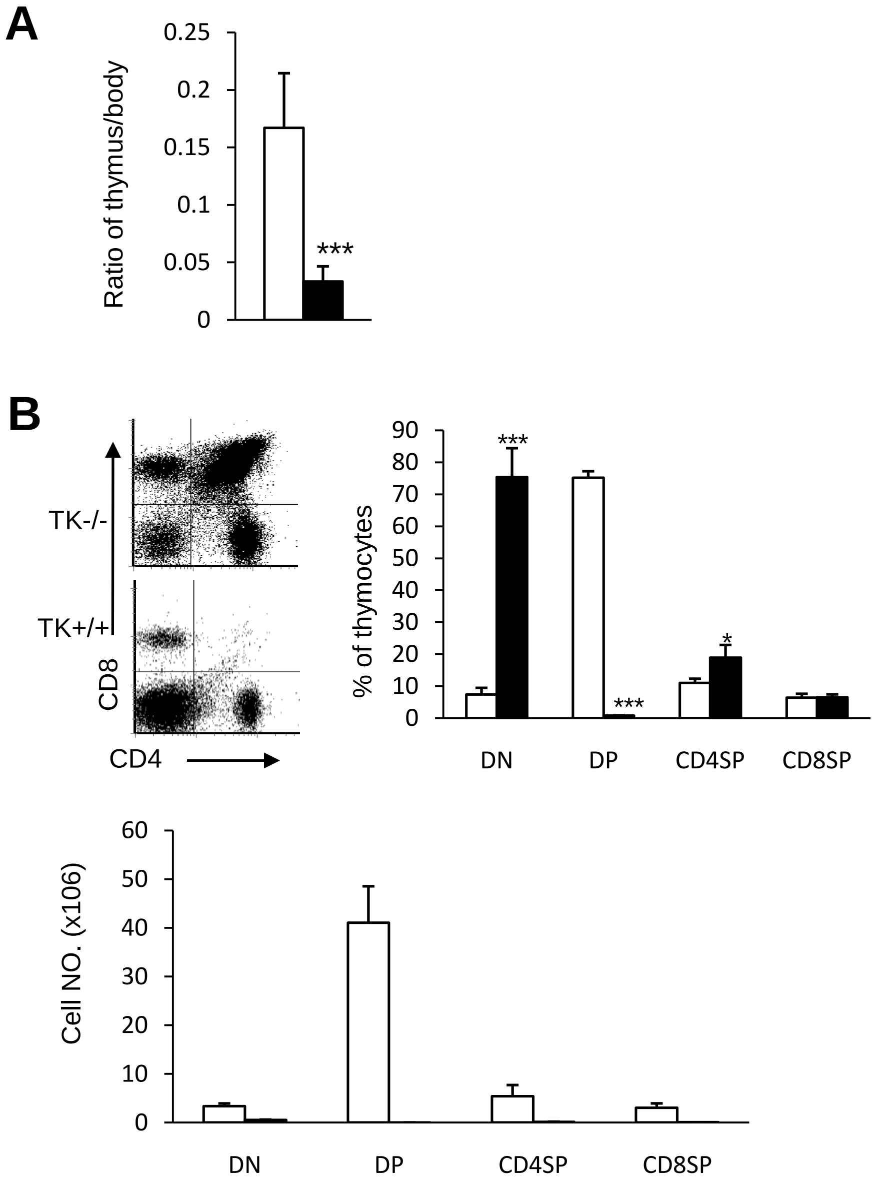

Suppl Fig 2. Thymocytes in FSP1$^+$ cells-deleted mice.

A, The ratio of the thymus weight to body weight in GCV-treated TK$^-$ and TK$^+$ mice were shown. **B**, Representative FACS and frequencies of thymocyte subsets in GCV-treated TK$^-$ (white bars) and TK$^+$ (black bars) mice.

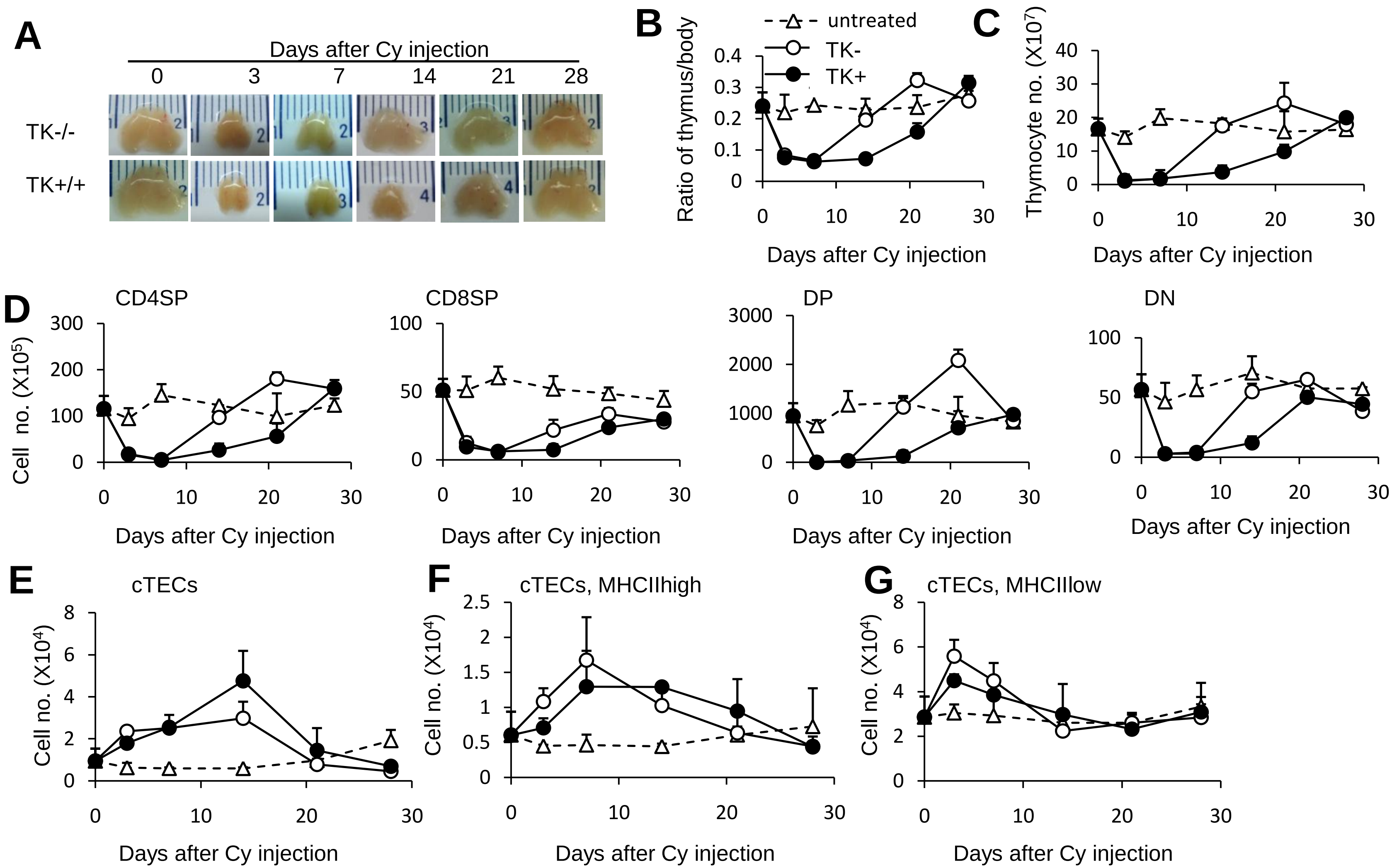

Suppl Fig 3. Changes of thymocytes and cTECs in Cy-induced thymus regeneration in FSP1⁺ cells-deleted mice. Cyclophosphamide (Cy)-induced thymus regeneration model was established in TK^- and TK^+ mice. During the course of thymus recovery, TK^- and TK^+ mice were treated with GCV for 14 days to delete proliferating $FSP1^+$ cells. **A,** Representative photographs of the thymus in Cy and GCV treated TK^- and TK^+ mice at indicated time points. The recovery curve of ratio of thymus weight to body weight (**B**) and total thymocyte number (**C**) of untreated (triangle), TK^- (open cycle) and TK^+ (closed cycle) after Cy injection. **D**, The recovery curve of thymocyte subsets including $CD4^+CD8^-$, $CD4^-CD8^+$, $CD4^+CD8^+$, and $CD4^-CD8^-$ cells of untreated, TK^- and TK^+ mice were shown. Recovery curve of cell number of cTECs (**E**), $MHCII^{high}$ (**F**) and $MHCII^{low}$ (**G**) cTECs in untreated, TK^- and TK^+ mice at various time points.

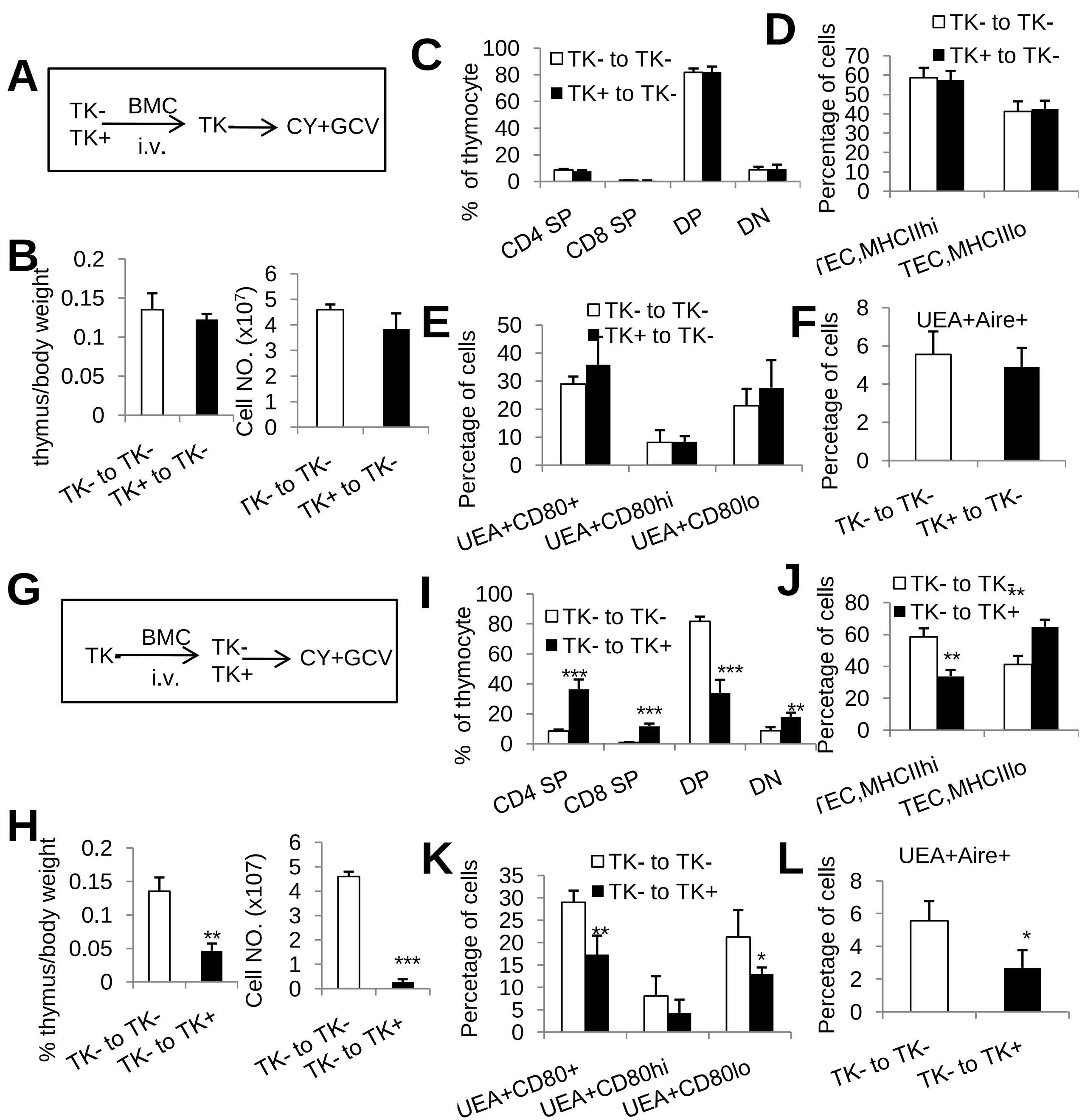

Suppl Fig 4. Non-hematopoietic FSP1⁺ cells play a critical role in thymus regeneration.

A, Full chimeric mice were generated by transplanting either TK^- and TK^+ BMCs to lethally irradiated TK- mice. By 8 weeks after transplantation, recipient mice were treated with Cy to induce thymus damage and with GCV for 14 days to delete $FSP1^+$ cells. **B**, The ratio of thymus weight to body weight and total cell number in mice received TK^- and TK^+ BMCs. **C**, Frequencies of thymocyte subsets in mice received TK^- and TK^+ BMCs were shown. **D**, The percentage of $MHCII^{high}$ and $MHCII^{low}$ TECs in recipients received TK^- and TK^+ BMCs. **E** and **F**, Frequencies of CD80+, $CD80^{hi}$, $CD80^{lo}$ and Aire on mTECs of mice received TK^- and TK^+ BMCs. **G**, Full chimeric mice were generated by transplanting TK^- BMCs to lethally irradiated either TK^- and TK^+ mice. **H**, The ratio of thymus weight to body weight and total cell number in TK^- and TK^+ recipients. **I**, Frequencies of thymocyte subsets in TK^- and TK^+ recipients were shown. **J**, The percentage of $MHCII^{high}$ and $MHCII^{low}$ TECs in TK^- and TK^+ recipients. **K** and **L**, Frequencies of CD80, $CD80^{hi}$, $CD80^{lo}$ and Aire on mTECs of recipients were presented.
